# Psychological wellbeing in adult adoptees: current age and developmental tasks

**DOI:** 10.3389/fpsyg.2023.1190147

**Published:** 2023-06-02

**Authors:** Sandra Melero, Laura Verdugo, Yolanda Sánchez-Sandoval

**Affiliations:** ^1^Department of Psychology, University of Cádiz, Puerto Real, Spain; ^2^Biomedical Research and Innovation Institute of Cadiz (INiBICA), Research Unit Puerta del Mar University Hospital, Cádiz, Spain

**Keywords:** adult development, wellbeing, adoption, developmental tasks, mediation

## Abstract

**Introduction:**

Studies about adult adoptees are normally focused on the differences in adjustment difficulties between them and non-adoptees. However, there has been less research about adoptees' positive and developmental adjustment in adulthood. The aim of this study is to test a model of the mediating role of adoptees' achievement of adulthood tasks in the relationship between current age and psychological wellbeing.

**Materials and methods:**

The sample consisted of 117 adults who were adopted as children by Spanish families. Their current mean age is 28.3 years. Participants underwent an interview and completed Ryff's Psychological WellBeing Scales.

**Results:**

Findings show that current age is directly and negatively related to psychological wellbeing [*c*′ = −0.039, 95% CI (−0.078, −0.001)], and the relationship between these variables is mediated by adoptees' achievement of adulthood tasks [indirect effect = 0.035, 95% CI (.014, 0.059)].

**Discussion:**

The findings support traditional theories about transitioning to adulthood, and adds relevant information about this transition in adoptees. Moreover, this work indicates a new way of assessing adoption success, based on long-term measures and normative variables. Services providers should account for the importance of supporting young people on their life transitions and promoting their wellbeing, especially among those who started from disadvantaged contexts.

## 1. Introduction

Adoption is a measure to provide a permanent and nurturing family to children whose family of origin is not able to offer them a secure context (Brodzinsky and Smith, 2019). Previous studies show the potential benefits of adoption in comparison to other child-protection measures such as foster care (Teyhan et al., 2018; DeLuca et al., 2019; Cáceres et al., 2021). Most of the studies about adoption have been carried out with children and adolescents, and mostly using parent-reported data. However, during the last decades there has been a growing interest in the study of long-term outcomes in adulthood. In this paper, we address the transition to adulthood in a group of Spanish adoptees in relation to their psychological wellbeing. As the life course perspective emphasizes, no stage of life can be understood in isolation from others, but that development is lifelong (Johnson et al., 2011).

The timing of transitions and their implications have long been a central concern for life-course analysts. Every life transition implies the need for adjustment to changes and the new roles related to them. Among life transitions, the transition to adulthood is particularly relevant for its challenges and singularity (Schulenberg and Schoon, 2012), especially for adoptees because they have some additional tasks to accomplish (Brodzinsky et al., 2014): new exploration of the meaning and implications of adoption, search for origins, or facing parenthood lacking information about the own genetic history. During emerging adulthood, adoptees continue to perform important psychological work related to the gathering of adoption-related information (Wrobel and Grotevant, 2019). As these authors highlight, in contrast to adolescence, in this life stage they can manage information about their adoption, or negotiate relationships within the adoptive kinship network, independently of their parents. Therefore, this transition to adulthood can have an additional personal impact for the adopted individuals because early adversity is associated to changes in their brain structure (Mackes et al., 2020), and it also might lead to a higher sensitivity to stress later on, when adoptees have to face adult life (McCrory et al., 2017).

Considering the changes and new responsibilities in people's lives, young adulthood may be a period of instability. A successful adulthood may be reached in several ways in terms of tasks, difficulties, and influences. Previous works state that there are certain life goals to achieve that are important to adult success (Layard et al., 2014; Mayseless and Keren, 2014). However, it is important to consider the large amount of variables such as historical changes that took place in the last decades, such as the decrease of the importance of marriage, the feminism role in our society, or changes in the labor market due to technology development (Estes and Sirgy, 2019).

In relation to the new roles, authors like Havighurst (1972) have defined the transition to adulthood in terms of tasks that people should accomplish in that life stage. These tasks (or “markers”) can include: finishing education, getting a job, assuming civic responsibilities, or other goals associated with relationships and family, like finding a stable social group, choosing a romantic partner, becoming a parent, being responsible for a home, etc. (Havighurst, 1972; Salmela-Aro et al., 2012; Schulenberg and Schoon, 2012). Common points among before mentioned authors are: getting a job, having a partner, becoming a parent, being responsible for a (own) home, and having a stable social group. However, the construct of adulthood tasks suffered an attempt of deconstruction during the last decade so that, currently, the achievement of some of these tasks is not considered necessary to succeed in the transition to adulthood (Schulenberg and Schoon, 2012).

The above-mentioned attempt of deconstruction should be considered from the viewpoint of social and historical changes, such as the reconceptualization of marriage or the labor market (Schulenberg and Schoon, 2012; Estes and Sirgy, 2019), and the current new demands and instability in young adulthood (Bonnie et al., 2015). However, the process of going through and achieving the previously mentioned goals has been linked to higher wellbeing (Ryff, 2014). Overall, the performance of these tasks should be taken into account with regard to adjustment and wellbeing.

According to Ryff (2014), people enjoy psychological wellbeing when they achieve balanced development and satisfaction in the following six life domains: purpose in life, autonomy, personal growth, environmental mastery, positive relations with others, and self-acceptance. However, multiple variables influence the development of wellbeing, and different authors have created models to illustrate this influence (Campion and Nurse, 2007; Thompson and Marks, 2008; Layard et al., 2014).

Campion and Nurse (2007) developed a dynamic model of wellbeing, which reflects the influences of personal, social and environmental risk factors (e.g., poor health, addictions or unemployment) for wellbeing, and the need to increase protective factors (i.e., relationships, physical activity, and confidence). Thompson and Marks (2008) developed another model that reflects dynamic and bidirectional influences between wellbeing, external conditions and personal resources, which both play an important role in wellbeing. Layard et al. (2014), using measures of life satisfaction as an indicator of wellbeing, developed a model including variables from the preceding stages, that is, childhood and adolescence. According to these authors, a useful model must combine variables from adult outcomes (economic, social, and personal) and influences of past characteristics. They estimated such influences both through past and present variables. However, childhood characteristics seem to have limited predictive power for adult life satisfaction/wellbeing. Supporting this model, it has been demonstrated that behavioral and emotional difficulties during adolescence have a direct negative effect on wellbeing in adult adoptees (Sánchez-Sandoval et al., 2020). These authors also found that child behavioral and emotional difficulties are directly related to the same types of difficulties in adolescence, but the relationship between childhood difficulties and adult wellbeing is only indirect through the presence of those difficulties in adolescence.

Previous studies about adopted children and adolescents revealed that they scored slightly lower than non-adoptees in positive variables of wellbeing (Moreno et al., 2016; Paniagua et al., 2020). Other works also showed that adoptees scored higher in self-esteem and life satisfaction than adolescents living under other protection measures such as residential care or foster care (Sánchez-Sandoval, 2015; Moreno et al., 2016).

Concerning adoptees' wellbeing, numerous pre-adoptive factors have been related to children's lower wellbeing, such as institutionalization prior to adoption, abuse, or neglect; but the most studied one is age at adoption (Vandivere and McKlindon, 2010). As well as these pre-adoptive aspects, post-adoption issues may affect children's wellbeing. Findings suggest that adoption enhances children's development and wellbeing because their adoptive home environments are more stable than those of children who remain in foster care (Zill and Bramlett, 2014).

Despite the importance of analyzing adjustment related to positive variables and wellbeing during adulthood, less research on adoption has been carried out with adult adoptees. According to the results of the systematic review performed by Melero and Sánchez-Sandoval (2017), most of the research on adult adoptees is based on a perspective of difficulties, and there are only a few works that analyze positive variables. It is important to clarify that most of the previous studies, not only in adulthood but also during the whole lifespan, find more difficulties among the group of adoptees if they are compared to non-adoptees (Askeland et al., 2017; Melero and Sánchez-Sandoval, 2017; Corral et al., 2021). Oke et al. (2015) included both positive and symptomatological variables, concluding that some adult adoptees had poor wellbeing.

An important variable to take into account as an influence for psychological wellbeing is age. Previous findings are inconclusive when considering a positive or negative relationship between these two variables. On the one hand, research has shown that psychological wellbeing tends to be higher in young and midlife adults than in older ones (Ryff and Keyes, 1995; Keresteš et al., 2012). Young adults tend to see themselves as improving over time, while older adults perceive their decline (Ryff, 2014). On the other hand, other studies found a positive relationship between age and psychological wellbeing (Archer et al., 2015). Lastly, another work revealed no correlation between age and psychological wellbeing in a specific sample of adult adoptees (Wall, 2011). However, it is important to consider that cultural context may imply differences in age-related outcomes (Karasawa et al., 2011).

The third decade of life seems to be the most decisive concerning wellbeing and life transitions (Salmela-Aro et al., 2012), although the achievement of the tasks implied in transition to adulthood varies depending on people and their resources (Schulenberg and Schoon, 2012). In line with this, Salmela-Aro et al. (2012) identified different role patterns in young adulthood that are related to wellbeing: on-time and postponed. Individual differences in the achievement of tasks are greater around the mid-20s and after the mid-30s (Schulenberg and Schoon, 2012). These authors indicated that people who had achieved multiple tasks by the age of 25 had greater wellbeing. Hence, a failure to achieve tasks by a certain age may lead to lower rates of wellbeing (Salmela-Aro et al., 2012).

According to Ryff (2014) summary of researches, several variables influence psychological wellbeing outcomes during adulthood. Some of them are related to the markers or tasks of transition to adulthood. Marriage has a positive influence on some dimensions of wellbeing (Nikolaev, 2018), and marital satisfaction is a positive predictor of some of the dimensions of psychological wellbeing. Thomas et al. (2017) state that there are different possible results when researching the effect of marriage over wellbeing considering gender. Additionally, a study with adult adoptees indicate no significant relationship between marital status and psychological wellbeing (Wall, 2011).

Concerning issues of economy and employment, employed people report higher wellbeing than unemployed people (Nikolaev, 2018; D'Agostino et al., 2019). However, the relationship between employment and wellbeing seems to be more important in mid adulthood than in early or later adulthood (Lansford, 2018). A higher level of income may lead to greater wellbeing. As the basic needs are met, and people have access to resources like better education, health, or other services, their perceptions of wellbeing increase. However, this is not a universal effect, as it is less significant at very high levels of income. Many other factors may also influence the relationship between income and wellbeing, such as job satisfaction (Viñas-Bardolet et al., 2020). A positive relationship between income and psychological wellbeing was also found in adult adoptive populations (Wall, 2011), and also unemployment have been related to higher vulnerability in this population (Golm et al., 2020).

Prior studies indicate that the association between parenting and wellbeing seems to depend on the analysis performed and the comparison group (Lansford, 2018). Current positive parenting experiences are directly related to positive wellbeing (Shin An and Cooney, 2006). Regarding relations with parents, García-Mendoza et al. (2017) found no differences in wellbeing between young adults who lived with their parents and those who did not, but the study of D'Agostino et al. (2019) reported that living with parents decreased life satisfaction. In line with this, life satisfaction seems to decrease after age 35 for those still living with their parents (Nikolaev, 2015). Family dynamics as a whole are a significant predictor of overall psychological wellbeing in adult adoptees (Wall, 2011).

Regarding education, results about wellbeing are inconclusive. Some earlier research showed a positive relationship between education and wellbeing (Keresteš et al., 2012; Nikolaev, 2018). However, other works indicated a neutral or negative relationship between education and wellbeing. A higher education may improve the availability of job options, the management of resources, health, social networks, attractiveness in relationships, and parenting quality. However, a higher education may also imply negative consequences, like more responsibility at work, longer working hours, more stress, and higher expectations that may lead to less satisfaction (Nikolaev, 2018). A positive relationship between educational level and wellbeing has also been found in adult adoptees (Wall, 2011).

Other variables also appear to be related to wellbeing in adulthood. Gender differences seem to be more important in other life stages, like adolescence or late adulthood. However, some authors found that women scored higher than men in wellbeing (Ryff, 2014), or some dimensions such as positive relations and personal growth (Karasawa et al., 2011; Matud et al., 2019). In contrast, other studies reported that men scored higher than women in almost all the subscales of psychological wellbeing (Shin An and Cooney, 2006), or in some of them, such as autonomy and self-acceptance (Karasawa et al., 2011). However, prior research found no gender differences in wellbeing when gender interacts with age, with both men and women showing similar scores as they grow older (Karasawa et al., 2011). Social support is also related to wellbeing, as a significant and positive predictor of all the dimensions of psychological wellbeing. Its influence is similar in males and females (Keresteš et al., 2012).

Most of the presented research provides information about the relationship of some variables with wellbeing outcomes during adulthood. Some of them are markers or tasks of transition to adulthood. However, there are little works with a wide and comprehensive approach, incorporating age and most of these tasks at the same time. The current study analyzes the relationship between age and the achievement of the main tasks related to transition to adulthood and psychological wellbeing during early adulthood, in a group of adult adoptees.

**Figure 1:**
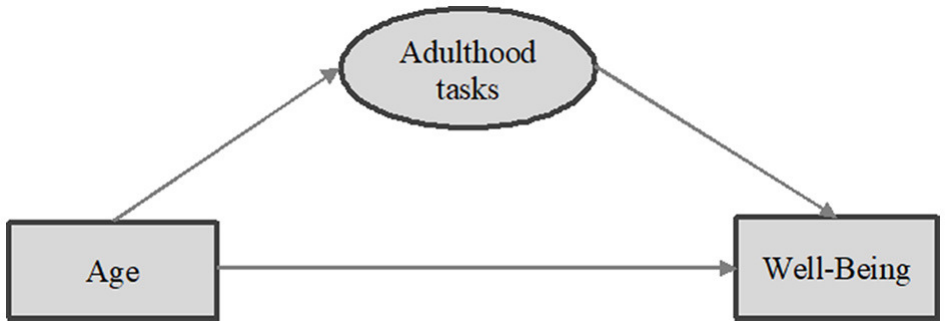
Hypothesized model.

The main aim of this study is to test a theoretical structural model, proposing a direct effect of current age and a mediating effect of the accomplishment of adulthood tasks on the psychological wellbeing of adult adoptees (see Figure 1). This model draws on the classical theories of development (e.g., Havighurst, 1972) and other studies (Salmela-Aro et al., 2012; Schulenberg and Schoon, 2012) although they did not refer specifically to adopted samples. According to the model, current age has a direct effect on psychological wellbeing. Additionally, the achievement of adulthood tasks would play a mediating role in the association between current age and psychological wellbeing. In relation to these overall aims, we tested the following hypotheses: (1) There is a connection between current age and the level of psychological wellbeing. (2) That relationship would be influenced by the accomplishment of certain adulthood tasks, like getting a job or becoming a parent.

## 2. Methods

### 2.1. Participants

One hundred seventeen adult Spanish adoptees (around 50% males) took part in this study. All participants had been adopted domestically from care, they were not international adoptions. The sample is part of a longitudinal study with adoptive families. There were three assessments: Wave 1 (W1) in 1995 (*n* = 394), Wave 2 (W2) in 2001 (*n* = 273), and Wave 3 in 2016–2018 (*n* = 117). The current study belongs to the third wave. At W1, there was an attempt of contacting the whole amount of families that adopted a child in the geographical area between 1987 and 1993, years between the legal regulations of adoption and the beginning of the project. The other two waves had the same sampling strategy.

Participants' age at W3 ranged from 23 to 44 years (*M* = 28.36, *SD* = 4.56). Their mean age at adoption was 1.92 years (*SD* = 3.14), and most of them were adopted before 1 year of age (56%). As shown in Table 1, 62% of the sample had completed some kind of high school education, and 35% of them were still studying. Additionally, 54% of the participants were working, 47% already had their own homes, 31% had children, and 67% had a stable romantic relationship [considered when one of the following criteria are met: being involved in their relationships for more than 12 months (93%) and/or were married or cohabitating (62%)].

**Table 1:** Sample characteristics.

**Characteristics (*****n*** = **117)**		***n***	**%**
**Individual**			
Gender	Male	59	50
	Female	58	50
Age (in years)	23–29	87	74
	≥30	30	26
Disability		12	10
Educational level	Primary school	13	11
	High school	73	62
	College	31	26
Currently studying		41	35
**Tasks and adjustment**			
Romantic relationship		79	67
Children		36	31
Job		63	54
Own home		55	47
Stable support		103	88

There were no missing data in this sample.

### 2.2. Instruments

#### 2.2.1. Adoption and life trajectories interview

We designed a semi-structured interview to collect participants' data on diverse life domains: academic achievement, career path, health (physical and mental), family situation, adoption trajectory, stressful life situations, social support, and personal resources. This interview included closed questions about the accomplishment of the adulthood tasks, like having a partner (e.g., *Are you involved in a stable romantic relationship?*) to which participants had to answer “Yes” or “No.” The score of “adulthood tasks” ranges from 0 to 5 and is made up of the sum of the affirmative answers in task accomplishment: having a partner, having children, living independently from the parents' home, having a good source of support and having a job. This kind of score has been used previously referring to developmental tasks in adulthood (Schulenberg et al., 2004; Piotrowski et al., 2020). These authors proposed an additive model consisting of the mean score of success in the developmental tasks. Success in as many as tasks as possible is considered the sole predictor of wellbeing. Summing the scores of yes/no questions has also been used with other psychological constructs, like cumulative risk (Bry et al., 1982) or adverse childhood experiences (McCrory et al., 2015; Deschênes et al., 2018).

#### 2.2.2. Psychological WellBeing Scales (Ryff and Keyes, 1995)

We used the Spanish short version (Díaz et al., 2006). It has 29 items rated from 1 (*Completely disagree*) to 6 (*Completely agree*). This scale includes a global mean score and the mean scores for the six subscales of the components of wellbeing: Purpose in life, Autonomy, Personal growth, Environmental mastery, Positive relations with others, and Self-acceptance. In this study, we only used the global mean score because of our focus on general wellbeing. The scale had excellent reliability (α = 0.91).

### 2.3. Procedure

First, we contacted families from the previous waves. We then scheduled appointments for the interview and the completion of the protocols. Interviews took place in the participants' homes or other places they considered comfortable, and lasted between one and two hours. Before the interview, all participants read and signed an informed consent. In addition, the Bioethics Committee of the University of Cádiz approved the project.

Due to the longitudinal design of this project, it is important to examine attrition across waves. In this case, we assessed the attrition between the first two waves and the third one finding that it was not systematic regarding some variables collected in earlier assessments (W1 and W2). First, we focused on the sociodemographic characteristics of the sample: gender proportions, age at adoption, simple or multiple adoption (adopted alone vs. adopted with siblings), ethnicity, disability, initial harshness (adverse childhood experiences prior adoption), educational level of the family, institutionalization, birth parents' substance use, maltreatment before adoption, family structure, family profession, mental disease or deficiency in biological parents, relation with the adoptee prior to adoption, and satisfaction with adoption at W1. None of them was significant.

We also compared participants and non-participants at Wave 3 according to measures of psychological adjustment from either Wave 1 or Wave 2. First, we used the Rutter Revised Parent Scale (W1 and W2), which assesses child emotional and behavioral problems. Most of the comparisons did not show significant differences between participants and non-participants at Wave 3, except for hyperactivity from Wave 1 [*t*_(271)_ = 2.528, *p* < 0.05; higher mean score for participants].

In addition, we compared participants using measures of self-esteem and life satisfaction from Wave 2. No significant differences were found. More information about these measures can be consulted in (Sánchez-Sandoval and Palacios, 2012; Sánchez-Sandoval, 2015).

### 2.4. Data analysis

Data analysis was carried out using the Statistical Program for the Social Sciences (SPSS). First, descriptive and correlation analyses were performed. Then, a model of the direct and indirect effects of current age as a continuum on psychological wellbeing was tested (see Figure 1). We examined whether the accomplishment of adulthood tasks (*M*) mediated the relationship between current age (*X*) and wellbeing (*Y*).

Before the mediation analysis, the data were checked for distribution normality with the skewness and kurtosis values. The skewness and kurtosis scores were −0.76 and 0.26 for wellbeing, −0.06 and −0.83 for adulthood tasks, and 1.35 and 1.68 for current age, respectively. These values are adequate for performing the analysis (skewness < 2, kurtosis < 7). The mediation effect of current age was tested with PROCESS for SPSS developed by Hayes (2017). A bootstrapping procedure was used to test the significance of the indirect effect. We also used a 95% confidence interval (CI) to test the mediator effect. If the interval does not include zero, the mediation effect is significant. Finally, the Sobel test was also calculated.

## 3. Results

### 3.1. Preliminary descriptive analyses

First, we compared wellbeing mean score with the theoretical mean of the scale (values range from 1 to 6, so 3.5 is the value). Findings show that adoptees on the sample score significantly higher than the theoretical mean, so their wellbeing is higher than the mean [*t*_(116)_ = 14.561, *p* < 0.01]. After that, we compared the means of every variable included in the model by gender. There were no significant differences between males and females in the number of accomplished adulthood tasks [*t*_(115)_ = −1.564, *p* > 0.05]; current age [*t*_(115)_ = 0.235, *p* > 0.05]; or psychological wellbeing [*t*_(115)_ = −1.763, *p* > 0.05]. Then, comparisons were made between people who achieved every developmental task and those who did not. Findings show that people who have a job, a stable relationship and a stable social network have higher levels of psychological wellbeing than those who do not, with moderate to high effect sizes (see Table 2).

**Table 2:** Differences in wellbeing concerning the achievement of every developmental task.

**Developmental task**	**Achieved**	**Non achieved**	***t***	***d***
Job	4.85	4.42	−2.82^**^	0.522
Stable relationship	4.78	4.40	−2.28^*^	0.450
Parenthood	4.48	4.73	1.27	0.290
Living independently	4.61	4.70	0.56	0.104
Stable social network	4.82	3.40	−6.84^***^	1.948

* *p* < 0.05.

** *p* < 0.01.

*** *p* < 0.001.

Dividing the sample into two groups, and comparing them both in the studied variables revealed no significant differences in psychological wellbeing between participants under or over 30 (*t* = 1.222, *p* > 0.05, *d* = 0.259). However, the variables related to developmental tasks (see Table 3) have significantly higher values among older participants (total tasks; *t* = −5.001, *p* > 0.05, *d* = 1.059). In addition, the mean ages of participants who achieved and not achieved developmental tasks were checked, finding significant differences between them. See Table 4 for more detailed results.

**Table 3:** Results in an age-divided sample.

	**Age**		**Chi-square**	**Phi**
	<**30**	≥**30**		
**Job**				
Yes	42	21	4.23^*^	0.19^*^
No	45	9		
**Stable relationship**				
Yes	53	26	6.74^*^	0.24^*^
No	34	4		
**Parenthood**				
Yes	15	21	29.14^*^	0.49^*^
No	72	9		
**Living independently**				
Yes	40	22	6.70^*^	0.23^*^
No	47	8		
**Stable social network**				
Yes	77	26	0.07	0.02
No	10	4		

* *p* < 0.05.

** *p* < 0.01.

**Table 4:** Mean ages of achievers and non-achievers of developmental tasks.

	**Yes**	**No**	***t***	***d***
Job	29.49	27.04	3.07^*^	0.55
Stable relationship	29.15	26.71	3.27^*^	0.55
Parenthood	32.25	26.63	5.77^*^	1.49
Living independently	29.89	26.64	4.18^*^	0.75
Stable social network	28.37	28.39	0.06	0.01

** *p* < 0.01.

Table 5 displays the means, standard deviations, and correlations between the variables in the study's estimated model. We found positive significant correlations between psychological wellbeing and the accomplishment of adulthood tasks. The achievement of adulthood tasks also correlated positively with current age. No significant correlation was observed between current age and psychological wellbeing (*r* = −0.021, *p* > 0.05).

**Table 5:** Correlations and mean scores.

	**Wellbeing**	**Adulthood tasks**	**Current age**
**Wellbeing**			
Adulthood tasks	0.239^*^		
Current age	−0.021	0.536^*^	
Mean	4.67	2.91	28.34
*SD*	0.84	1.30	4.79

** *p* < 0.01.

### 3.2. Model testing

**Figure 2:**
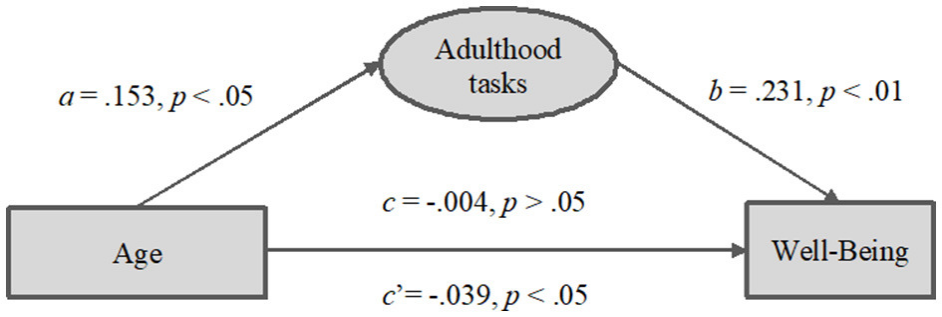
Simple mediation using the mediating effect of adulthood tasks on the relationship between age and wellbeing.

Results of a simple mediation analysis indicated that current age is indirectly related to psychological wellbeing through its relationship with the achievement of adulthood tasks. Figure 2 and Table 6 show the results of the mediation analysis, including information about the coefficients (Coeff.). Considering the Sobel test (*Z* = 3.016, *p* = 0.002), the model showed a significant mediator effect. Current age had a direct positive effect on the participant's achievement of adulthood tasks (*a*) [Coeff. = 0.153; 95% CI (0.108, 0.197), *p* < 0.001]. In addition, the effect of age on the prediction of wellbeing was significant [*c*′; Coeff. = −0.039; 95% CI (−0.078, −0.001), *p* < 0.05]. The achievement of adulthood tasks significantly predicted psychological wellbeing [*b*; Coeff. = 0.231; 95% CI (0.093, 0.369), *p* < 0.01]. The mediation analysis revealed that the total and direct effects of current age on the level of psychological wellbeing were, respectively, −0.004, and −0.039. A 95% bias-corrected confidence interval based on 10000 bootstrap samples indicated that the indirect effect of current age on wellbeing through the accomplishment of adulthood tasks (*ab* = 0.035) was amply above zero (0.014, 0.059). Moreover, Table 6 shows some effect size measures for the model (standardized indirect effects and coefficients, *R*^2^, *K*^2^, proportion of indirect effect on the direct effect).

**Table 6:** Mediational model coefficients.

**Predictors**		**Consequent**					
		**Adulthood tasks**			**Wellbeing**		
		**Coefficient**	***SE***	***p*** **-value**	**Coefficient**	***SE***	***p*** **-value**
Age		*a* = 0.153	0.022	< 0.001	*c*′= −0.039	0.019	< 0.05
Adulthood tasks		–	–	–	*b* = 0.231	0.069	< 0.01
Constant		−1.411	0.646	< 0.05	5.096	0.493	< 0.001
		*R*^2^ = 0.286			*R*^2^ = 0.088		
		*F*_(1, 115)_ = 46.268, *p* < 0.001			*F*_(2, 114)_ = 5.544, *p* < 0.01		
Effect size measures		Partially standardized indirect effects of age			0.041		
		Completely standardized indirect effects of age			0.188		
		Standardized coefficients			*a* = 0.535, *b* = 0.351, *c*′= −0.209, *c* = −0.021		
		Ratio of the indirect effect of the direct effect			0.896		
		*K*^2^ (ab over maximum ab)			0.593		

ab = indirect effect.

As shown in Figure 2 and Table 6, the presented indexes (*ab* and *c*′) have opposite signs, making this mediation inconsistent (Warner, 2013). According to this author, this fact does not imply an absence of mediation, but a suppressor effect of the indirect effect on the direct effect. In this particular case, one might refer to competitive mediation, as stated by Zhao et al. (2010), because the direct effect points in the opposite direction than the indirect effect. The main consequence of this kind of mediation is that the total effect is close to zero and, consequently, non-significant.

Current age negatively predicted psychological wellbeing, but when including the achievement of adulthood tasks in the model, this total effect may change due to the positive indirect effect of the achievement of adulthood tasks. In other words, in our data, there is no direct relationship between getting older and a decrease on psychological wellbeing. The increase of age is only related to a lower psychological wellbeing in case of lack of mastery on certain adulthood developmental tasks.

## 4. Discussion

The purpose of this study was to test a model of the relationship between current age and psychological wellbeing, and the possible mediator effect of the achievement of adulthood tasks in this relationship. Results show a significant direct connection between current age and the level of psychological wellbeing. In addition, the indirect effect of the achieved tasks was significant in the association between these two variables, showing that task achievement plays an important mediator role. We also examined gender differences in the target variables. Findings showed no significant differences between males and females of this sample in wellbeing, current age, or the number of accomplished adulthood tasks.

Previous studies showed higher levels of wellbeing in young and midlife adults in comparison to older ages (Ryff and Keyes, 1995; Keresteš et al., 2012). Considering, sample's age it is difficult to see those data reflected. However, as shown previously they have good levels of wellbeing compared to the theoretical mean, so that does not contradict prior findings. Nevertheless, the age between 20 and 30 years is critical for the attainment of adulthood tasks, which are also related to wellbeing (Salmela-Aro et al., 2012). It is important to take individual differences into account in this path (Schulenberg and Schoon, 2012). Considering that the age of the sample of this study ranged from 23 to 44 years, with a mean around 28, it could be concluded that most of the participants are undergoing this critical period. This might be one of the reasons why age played an important role in this study. That age period (from 20 to 30) is also characterized by the presence of more distress and instability because of the need to adjust to new roles (Bonnie et al., 2015), and the implications from early life experiences for adoptees (McCrory et al., 2017; Mackes et al., 2020).

Prior studies also found a relationship between the achievement of adulthood tasks and psychological wellbeing (Shin An and Cooney, 2006; Ryff, 2014; Nikolaev, 2015, 2018; Lansford, 2018), and some highlight the importance of age in that association (Salmela-Aro et al., 2012; Schulenberg and Schoon, 2012). These latter studies revealed that young adulthood is a critical moment for the development or achievement of certain tasks, which will also influence psychological wellbeing.

On another hand, there has been a recent attempt to deconstruct the concept of adulthood tasks and their achievement. According to Schulenberg and Schoon (2012), the attainment of certain goals is considered unnecessary for a successful transition to adulthood. In this sense, pathways for the mentioned transition can be quite heterogeneous, and there is a large range of variability in the configuration of the tasks. There could be several explanations for this. One of them is the presence of historical, social, and cultural influences. From the historical point of view, many things have changed in the last decades, like the importance of marriage, women's role in society, or employment skills (Schulenberg and Schoon, 2012; Estes and Sirgy, 2019). Another important factor is the 21st century sociocultural perspective of young adulthood, which considers this life stage a critical period in development, more demanding in terms of information and economical resources, less predictable, a possible magnifier of inequality, unhealthy, and in need of support (Bonnie et al., 2015).

Finally, Salmela-Aro et al. (2012) revealed different profiles regarding adulthood tasks: slow starters, highly educated with family, highly educated without family, and traditional work and family. These authors conclude that people with greater wellbeing are those who completed high education and who have their own families. On the contrary, those who still have to achieve tasks related to career or family (slow starters, or highly educated without family) have lower levels of wellbeing. These results, together with those the present study, support the idea of classical theories about adulthood task accomplishment and its relationship to psychological wellbeing, despite the attempts of deconstruction.

This research was conducted with a group of adult adoptees. Adoptees' adjustment in adulthood has been traditionally measured through the presence of problems, but few works have considered a perspective of wellbeing (Melero and Sánchez-Sandoval, 2017). In addition, wellbeing in adoptees is normally predicted by taking into account child and adolescent difficulties, so, in this study, we wanted to use variables that are typically assessed in adulthood as another way of considering a successful adoption. In this particular case, we tried to assess not only the success of the adoption process, but also the long-term success of the adoption *per se*, referring to the achievement of a successful development during the lifespan. As adoption is a measure to protect children and provide them a secure context to improve their outcomes, one might think that a successful development is also an attainment on the adoption process.

In line with the aforementioned ideas, it is noteworthy that the adoptees in this research generally show fairly high scores in wellbeing. It means that respondents in this study on average agreed to all positive items, which also implies that their score is significantly higher than the theoretical mean. A possible explanation of this result concerns the change of their childhood social status when they were adopted. In these adoptees' particular case, they experienced a change in terms of opportunities when suitable families adopted them. Consequently, the assimilation of values and norms in these people might proceed from their adoptive families. It is also likely that social support and positive family dynamics in the adoptive families had a positive influence on adoptees' wellbeing. This work provides evidence that support ideas of the life-course academics; to explain these people's wellbeing in adulthood is necessary to look back and consider important life events from their childhood, such as adoption and prior experiences. At the same time, this study also confirms the permeability of human development considering the influence of positive experiences through the lifespan, like the accomplishment of some of the adulthood tasks.

### 4.1. Limitations

The present study has some limitations that should be reflected. It would be more appropriate to test the model presented in this work in a control group of non-adopted people with similar characteristics. This comparison would provide a more complete view of the situation. In addition, the omission of adoption-specific tasks in the model is a possible limitation to consider. Another drawback to take into account is the sample size. The longitudinal design of the study presented herein makes it difficult to achieve a larger sample. However, the current sample in W3 is representative of the sample of W1, considering the results of the attrition analyses. In addition, it is important to consider that previous research with adoptees also used relatively small samples (Oke et al., 2015; Balenzano et al., 2018).

### 4.2. Implications for future research

The study provides a new research approach to adoption from a different perspective: the long-term success of adoption as assessed by variables used in general population during adulthood. This will allow us to focus on the development process rather than on the difficulties *per se*. This new approach could also help to depathologize the concept of adoption and the development of psychological wellbeing in the people involved. This work supports the idea that there is a growing need to study adult adoptees from a positive perspective. For this purpose, both longitudinal and cross-sectional studies are necessary to consider not only their past but also their current characteristics. For future works, it will be relevant to include also the analysis of adoption-specific tasks to see their influence on the process of transitioning to adulthood.

It is also important to transfer research results to practice, so it is necessary to have the appropriate services not only to help adult adoptees, but also to understand the possible particularities of this group. According to Bonnie et al. (2015), interventions encounter three main problems: they are not coordinated, they do not have the adequate focus, and most of them are not based on evidence. These authors also stated that the future wellbeing of our society is related to the investments made in current young adults, especially the ones that belong to high-risk groups. They highlight that providing these youngsters with more opportunities concerning education, economy, social life, and health will improve their possibilities for successful adult development. So, a possible way to improve adoption success might be the design of interventions to provide support for adoptees' life transitions and to promote the development of their wellbeing, and probably, their health in general.

## Data availability statement

The raw data supporting the conclusions of this article will be made available by the authors, without undue reservation.

## Ethics statement

The study, involving human participants, was reviewed and approved by Bioethics Committee of the University of Cádiz. The patients/participants provided their written informed consent to participate in this study.

## Author contributions

All authors listed have made a substantial, direct, and intellectual contribution to the work and approved it for publication.

## References

[B1] Archer J. A. Lim Z. M. T. Teh H. C. Chang W. C. Chen S. H. A. (2015). The effect of age on the relationship between stress, well-being and health in a Singaporean sample. Ageing Int. 40, 413–425. 10.1007/s12126-015-9225-3

[B2] Askeland K. G. Hysing M. La Greca A. M. Aarø L. E. Tell G. S. Sivertsen B.. (2017). Mental health in internationally adopted adolescents: a meta-analysis. J. Am. Acad. Child Adolesc. Psychiatry 56, 203–213. 10.1016/j.jaac.2016.12.009 28219486

[B3] Balenzano C. Coppola G. Cassibba R. Moro G. (2018). Pre-adoption adversities and adoptees' outcomes: the protective role of post-adoption variables in an Italian experience of domestic open adoption. Child. Youth Serv. Rev. 85, 307–318. 10.1016/j.childyouth.2018.01.012

[B4] Bonnie R. J. Stroud C. Breiner H. (2015). Investing in the Health and Well-being of Young Adults. Washington, DC: The National Academies Press. 25855847

[B5] Brodzinsky D. M. Schechter M. Henig R. M. (2014). Soy Adoptado. La Vivencia de la Adopción a lo Largo de la Vida. Madrid: Editorial Grupo 5.

[B6] Brodzinsky D. M. Smith S. L. (2019). Commentary: understanding research, policy, and practice issues in adoption instability. Res. Soc. Work Pract. 29, 185–194. 10.1177/1049731518782647

[B7] Bry B. H. McKeon P. Pandina R. J. (1982). Extent of drug use as a function of number of risk factors. J. Abnorm. Psychol. 91, 273–279. 10.1037/0021-843X.91.4.273 7130523

[B8] Cáceres I. Moreno C. Román M. Palacios J. (2021). The social competence of internationally-adopted and institutionalized children throughout childhood: a comparative and longitudinal study. Early Child. Res. Q. 57, 260–270. 10.1016/j.ecresq.2021.07.002

[B9] Campion J. Nurse J. (2007). A dynamic model for wellbeing. Australas. Psychiatry 15, 24–27. 10.1080/10398560701701106 18027131

[B10] Corral S. Herrero M. Martín N. Gordejuela A. Herrero-Fernández D. (2021). Psychological adjustment in adult adoptees: a meta-analysis. Psicothema 33, 527–535. 10.7334/psicothema2021.98 34668466

[B11] D'Agostino A. Grilli G. Regoli A. (2019). The determinants of subjective well-being of young adults in Europe. Appl. Res. Qual. Life 14, 85–112. 10.1007/s11482-017-9582-z

[B12] DeLuca H. K. Claxton S. E. Dulmen M. H. M. (2019). The Peer relationships of those who have experienced adoption or foster care: a meta-analysis. J. Res. Adolesc. 29, 796–813. 10.1111/jora.12421 29938859

[B13] Deschênes S. S. Graham E. Kivimäki M. Schmitz N. (2018). Adverse childhood experiences and the risk of diabetes: examining the roles of depressive symptoms and cardiometabolic dysregulations in the Whitehall II cohort study. Diabetes Care 41, 2120–2126. 10.2337/dc18-0932 30072405 PMC6150425

[B14] Díaz D. Rodríguez-Carvajal R. Blanco A. Moreno-Jiménez B. Gallardo I. Valle C.. (2006). Adaptación española de las escalas de bienestar psicológico de Ryff. Psicothema 18, 572–577. 17296089

[B15] Estes R. J. Sirgy M. J. (2019). Global advances in quality of life and well-being: past, present, and future. Soc. Indic. Res. 141, 1137–1164. 10.1007/s11205-018-1869-4

[B16] García-Mendoza M. C. Parra Á. Sánchez-Queija I. (2017). Relaciones familiares y ajuste psicológico en adultos emergentes universitarios españoles. Behav. Psychol. 25, 405–417.

[B17] Golm D. Maughan B. Barker E. D. Hill J. Kennedy M. Knights N.. (2020). Why does early childhood deprivation increase the risk for depression and anxiety in adulthood? A developmental cascade model. J. Child Psychol. Psychiatry 61, 1043–1053. 10.1111/jcpp.13205 32026473 PMC8597399

[B18] Havighurst R. J. (1972). Developmental Tasks and Education. New York, NY: David McKay Company.

[B19] Hayes A. F. (2017). Introduction to Mediation, Moderation, and Conditional Process Analysis: A Regression-based Approach, 2nd ed. New York, NY: The Guilford Press.

[B20] Johnson M. K. Crosnoe R. Elder G. H. (2011). Insights on adolescence from a life course perspective. J. Res. Adolesc. 21, 273–280. 10.1111/j.1532-7795.2010.00728.x 21483644 PMC3072576

[B21] Karasawa M. Curhan K. B. Markus H. R. Kitayama S. S. Love G. D. Radler B. T.. (2011). Cultural perspectives on aging and well-being: a comparison of Japan and the United States. Int. J. Aging Hum. Dev. 73, 73–98. 10.2190/AG.73.1.d 21922800 PMC3183740

[B22] Keresteš G. Brković I. Kuterovac Jagodić G. (2012). Predictors of psychological well-being of adolescents' parents. J. Happiness Stud. 13, 1073–1089. 10.1007/s10902-011-9307-119187084

[B23] Lansford J. E. (2018). “A lifespan perspective on subjective well-being,” in Handbook of Well-Being, eds E. Diener, S. Oishi, and L. Tay (Salt Lake City: DEF Publishers). Available online at: https://nobascholar.com (accessed November 2021).

[B24] Layard R. Clark A. E. Cornaglia F. Powdthavee N. Vernoit J. (2014). What predicts a successful life? A life-course model of well-being. Econ. J. 124, F720–F738. 10.1111/ecoj.12170 25422527 PMC4240315

[B25] Mackes N. K. Golm D. Sarkar S. Kumsta R. Rutter M. Fairchild G.. (2020). Early childhood deprivation is associated with alterations in adult brain structure despite subsequent environmental enrichment. Proc. Natl. Acad. Sci. 117, 641–649. 10.1073/pnas.1911264116 31907309 PMC6955353

[B26] Matud M. P. López-Curbelo M. Fortes D. (2019). Gender and psychological well-being. Int. J. Environ. Res. Public Health 16, 3531. 10.3390/ijerph16193531 31547223 PMC6801582

[B27] Mayseless O. Keren E. (2014). Finding a meaningful life as a developmental task in emerging adulthood: the domains of love and work across cultures. Emerg. Adulthood 2, 63–73. 10.1177/2167696813515446

[B28] McCrory C. Dooley C. Layte R. Kenny R. A. (2015). The lasting legacy of childhood adversity for disease risk in later life. Health Psychol. 34, 687–696. 10.1037/hea0000147 25150540

[B29] McCrory E. J. Gerin M. I. Viding E. (2017). Annual research review: childhood maltreatment, latent vulnerability and the shift to preventative psychiatry - the contribution of functional brain imaging. J. Child Psychol. Psychiatry 58, 338–357. 10.1111/jcpp.12713 28295339 PMC6849838

[B30] Melero S. Sánchez-Sandoval Y. (2017). Mental health and psychological adjustment in adults who were adopted during their childhood: a systematic review. Child. Youth Serv. Rev. 77, 188–196. 10.1016/j.childyouth.2017.05.006

[B31] Moreno C. Ramos P. Rivera F. Jiménez-Iglesias A. García-Moya I. Sánchez-Queija I.. (2016). Los Adolescentes Españoles: Estilos de Vida, Salud, Ajuste Psicológico y Relaciones en Sus Contextos de Desarrollo. Resultados del Estudio HBSC-2014 en España. Health Behaviour in school-aged children. Madrid: Ministerio de Sanidad, Servicios Sociales e Igualdad.

[B32] Nikolaev B. (2015). Living with mom and dad and loving it….or are you? J. Econ. Psychol. 51, 199–209. 10.1016/j.joep.2015.08.009

[B33] Nikolaev B. (2018). Does higher education increase hedonic and eudaimonic happiness? J. Happiness Stud. 19, 483–504. 10.1007/s10902-016-9833-y33995163

[B34] Oke M. Groza V. Park H. Kalyanvala R. Shetty M. (2015). The perceptions of young adult adoptees in India on their emotional well-being. Adopt. Foster. 39, 343–355. 10.1177/0308575915611776

[B35] Paniagua C. Moreno C. Román M. Palacios J. Grotevant H. D. Rivera F.. (2020). Under the same label: adopted adolescents' heterogeneity in well-being and perception of social contexts. Youth Soc. 52, 1544–1568. 10.1177/0044118X19828081

[B36] Piotrowski K. Brzezińska A. I. Luyckx K. (2020). Adult roles as predictors of adult identity and identity commitment in Polish emerging adults: psychosocial maturity as an intervening variable. Curr. Psychol. 39, 2149–2158. 10.1007/s12144-018-9903-x

[B37] Ryff C. D. (2014). Psychological well-being revisited: advances in the science and practice of eudaimonia. Psychother. Psychosom. 83, 10–28. 10.1159/000353263 24281296 PMC4241300

[B38] Ryff C. D. Keyes C. L. M. (1995). The structure of psychological well-being revisited. J. Pers. Soc. Psychol. 69, 719–727. 10.1037/0022-3514.69.4.719 7473027

[B39] Salmela-Aro K. Ek E. Taanila A. Chen M. (2012). Role configurations in young adulthood, antecedents, and later wellbeing among Finns born in 1966. Longitud. Life Course Stud. 3, 228–242. 10.14301/llcs.v3i2.184

[B40] Sánchez-Sandoval Y. (2015). Self-perception, self-esteem and life satisfaction in adopted and non-adopted children and adolescents. Infancia Aprendiz. 38, 144–174. 10.1080/02103702.2014.996406

[B41] Sánchez-Sandoval Y. Melero S. López-Jiménez A. M. (2020). Mediating effects of social support in the association between problems in childhood and adolescence and well-being in adult domestic adoptees. J. Happiness Stud. 21, 1183–1198. 10.1007/s10902-019-00124-8

[B42] Sánchez-Sandoval Y. Palacios J. (2012). Problemas emocionales y comportamentales en niños adoptados y no adoptados. Clínica Salud. 23, 221–234. 10.5093/cl2012v23n1a?

[B43] Schulenberg J. E. Bryant A. L. O'Malley P. M. (2004). Taking hold of some kind of life: how developmental tasks relate to trajectories of well-being during the transition to adulthood. Dev. Psychopathol. 16, 1119–1140. 10.1017/S0954579404040167 15704830

[B44] Schulenberg J. E. Schoon I. (2012). The transition to adulthood across time and space: overview of special section. Longitud. Life Course Stud. 3, 164–172. 10.14301/llcs.v3i2.194 26473017 PMC4603838

[B45] Shin An J. Cooney T. M. (2006). Psychological well-being in mid to late life: the role of generativity development and parent-child relationships across the lifespan. Int. J. Behav. Dev. 30, 410–421. 10.1177/0165025406071489

[B46] Teyhan A. Wijedasa D. MacLeod J. (2018). Adult psychosocial outcomes of men and women who were looked-after or adopted as children: prospective observational study. BMJ Open 8, e019095. 10.1136/bmjopen-2017-019095 29439075 PMC5829744

[B47] Thomas P. A. Liu H. Umberson D. (2017). Family relationships and well-being. Innov. Aging 1, 1–11. 10.1093/geroni/igx025 29795792 PMC5954612

[B48] Thompson S. Marks N. (2008). Measuring Well-being in Policy: Issues and Applications. London: New Economics Foundation.

[B49] Vandivere S. McKlindon A. (2010). The well-being of U.S. children adopted from foster care, privately from the United States and internationally. Adopt. Q. 13, 157–184. 10.1080/10926755.2010.524871

[B50] Viñas-Bardolet C. Guillen-Royo M. Torrent-Sellens J. (2020). Job characteristics and life satisfaction in the EU: a domains-of-life approach. Appl. Res. Qual. Life 15, 1069–1098. 10.1007/s11482-019-09720-5

[B51] Wall D. (2011). The Impact of Family Dynamics on Psychological Well-being in Adult Adoptees. Baltimore, MD: Walden University.

[B52] Warner R. M. (2013). “Mediation,” in Applied Statistics: From Bivariate through Multivariate Techniques (Thousand Oaks, CA: Sage Publications), 645–687.

[B53] Wrobel G. M. Grotevant H. D. (2019). Minding the (information) gap: what do emerging adult adoptees want to know about their birth parents? Adopt. Q. 22, 29–52. 10.1080/10926755.2018.1488332 31485156 PMC6726404

[B54] Zhao X. Lynch J. G. Chen Q. (2010). Reconsidering Baron and Kenny: myths and truths about mediation analysis. J. Consum. Res. 37, 197–206. 10.1086/651257

[B55] Zill N. Bramlett M. D. (2014). Health and well-being of children adopted from foster care. Child. Youth Serv. Rev. 40, 29–40. 10.1016/j.childyouth.2014.02.008

